# Assessment of scintillation and Cherenkov imaging as beam shape verification method in CyberKnife® radiotherapy

**DOI:** 10.1002/acm2.14508

**Published:** 2024-09-06

**Authors:** Fengwei Cui, Tao Jin, Mingzhu Li, Lei Zhu, Xing Di, Huaguang Zhu

**Affiliations:** ^1^ CyberKnife Center Department of Neurosurgery Huashan Hospital Fudan University Shanghai China; ^2^ Neurosurgical Institute of Fudan University Shanghai China; ^3^ Department of Oncology The First Hospital of Hebei Medical University Hebei Medical University Shijiazhuang China; ^4^ Department of Radiation Oncology Physics and Technology Shandong Cancer Hospital and Institute Shandong First Medical University and Shandong Academy of Medical Sciences Jinan City Shandong Province China

**Keywords:** Cherenkov imaging, CyberKnife® radiotherapy, QA, scintillation imaging

## Abstract

**Purpose:**

The goal of this study is to assess the utility of Cherenkov imaging (CI) and scintillation imaging (SI) as high‐resolution techniques to measure CyberKnife® beam shape quantitatively at the irradiation surface in quality assurance (QA).

**Methods:**

The EMCCD camera captured scintillation and Cherenkov photons arising from 6 MV x‐ray dose deposition produced by the CyberKnife® VSI System. Two imaging methods were done at source to surface distance of 800 cm with the same field size, ranging from 10 to 60 mm using fixed cones and iris collimators. The output sensitivity and constancy were measured using the SI and CI, and benchmarked against an ionization chamber. Line profiles of each beam measured by optical imaging were compared with film measurement. Position shifts were introduced to test the sensitivity of SI and CI to small beam position deviations. To assess reproducibility, the beam measurements were tested three times on 5 consecutive days.

**Results:**

Both systems exhibited comparable sensitivity to the ionization chamber in response to fluctuations in CyberKnife® output. The beam profiles in SI matched well with the measured film image, with accuracy in the range of ± 0.20 and ± 0.26 mm standard deviation for the circle and iris field, respectively. The corresponding accuracy measured by CI is in the range of ± 0.25 and ± 0.33 mm, respectively. These are all within the tolerance recommended by the guidelines of CyberKnife® QA. The accuracy measured by SI and CI for 1 mm beam position shift within 0.21 and 0.45 mm tolerance, respectively. Repeatability measurements of the beam have shown a standard deviation within 0.94 mm.

**Conclusions:**

SI and CI techniques are tested to provide a valid way to measure CyberKnife® beam shape in this study. Meanwhile, the systematic comparison of SI and CI also provides evidence for the measurement methods selection appropriately.

## 1 INTRODUCTION

Stereotactic radiosurgery (SRS) is extensively used for the treatment of brain tumors due to its remarkable curative effect.^1^, ^2^, ^3^, ^4^ The CyberKnife® is a frameless SRS instrument involving a 6/10 MV linear accelerator (LINAC) mounted on a robotic arm. This system employs multiple narrow beams to deliver conformed and precise radiation doses to the target from different directions.^5^, ^6^, ^7^, ^8^ Verification of the accuracy of the CyberKnife® radiation beam, as shaped by fixed cones and variable aperture iris collimators, constitutes an essential QA component in CyberKnife® radiotherapy.^9^ High‐resolution techniques are needed for small radiation beam measurement used in CyberKnife® radiotherapy QA, in view of the lateral electronic disequilibrium and steep dose gradients that exist in these small fields.^10^, ^11^, ^12^, ^13^, ^14^, ^15^, ^16^ However, several conventional techniques have failed to meet the measurement requirements for CyberKnife® QA. While ionization chamber is considered the gold standard for radiation dose measurements, it is susceptible to volume‐averaging effects for small beamlet QA.^17^ It also does not permit high‐resolution dose measurement owing to the large size of individual ionization chambers.^18^ TLD and OSLD are unable to require 2D dose distribution and need for time‐intensive readout processing, therefore, they are primarily suitable for point‐dose.^19^, ^20^, ^21^ Film offered a high‐resolution method for CyberKnife® QA, however, it is relatively insensitive to low‐dose exposures and is intended for one‐time use.^22^, ^23^ Therefore, developing a robust, high‐resolution, and time‐saving solution for CyberKnife® QA measurements is warranted.

Nowadays, with the advancement of optical detection devices, optical imaging methods have been introduced and utilized for LINAC beam QA measurements, especially for 2D beam shape measurements from Cherenkov emission or scintillating target emission. Previous studies have indicated that the Cherenkov emission is proportional to the dose deposited by MV x‐ray.^24^, ^25^ Based on this, Miao et al. have been conducted to measure the 2D LINAC beam shape with CI.^26^ Additionally, Black et al. also verified the feasibility of applying CI to monitor the beam match line on solid water.^27^ Scintillation imaging provides a direct radiation beam size or direct surface dose value with accuracy comparable to film. Alexander et al. proposed to perform routine QA measurements on an MR‐Linac with the use of a scintillation imaging system.^28^ Similar system is carried out to image field profile and measure delivery rates used in pencil‐beam scanning proton therapy.^29^, ^30^ The Cherenkov‐excited fluorescence imaging was also applied to daily QA for different radiotherapy Linac. Ramish et al. demonstrated the feasibility of Cherenkov‐excited fluorescence imaging as a QA tool for beamlets in VMAT plans.^31^ Verification of mechanical‐imaging‐radiation isocenter coincidence and beam characteristics are also performed through Cherenkov‐excited fluorescence for daily QA on an MR‐Linac.^32^, ^33^, ^34^ Above all, Cherenkov and scintillation imaging have been demonstrated as useful methods for various QA procedures in traditional radiotherapy. However, past research usually focuses on optical imaging beam shapes by individual methods of CI or SI, and overlooks the antithesis between these two methods.

This study is motivated by the need to develop a QA tool for CyberKnife® which is real‐time, high spatial resolution, cost‐effective, easy to use, and can achieve measurement precision comparable to film. In this study, the CyberKnife® beam shapes are measured and analyzed quantitatively by CI and SI during solid water and GOS: Tb scintillation screen irradiation, respectively. The goal is to assess which, if any, of these optical methods are accurate enough to rely upon for routine QA procedures on CyberKnife® radiotherapy and thus provide a rapid, high‐resolution. and reliable solution to many routine QA procedures including MU response, beam shape measurements, position variation sensitivity, and measurements reproducibility.

## 2 METHODS AND MATERIALS

### 2.1 Experimental radiation delivery

**FIGURE 1:**
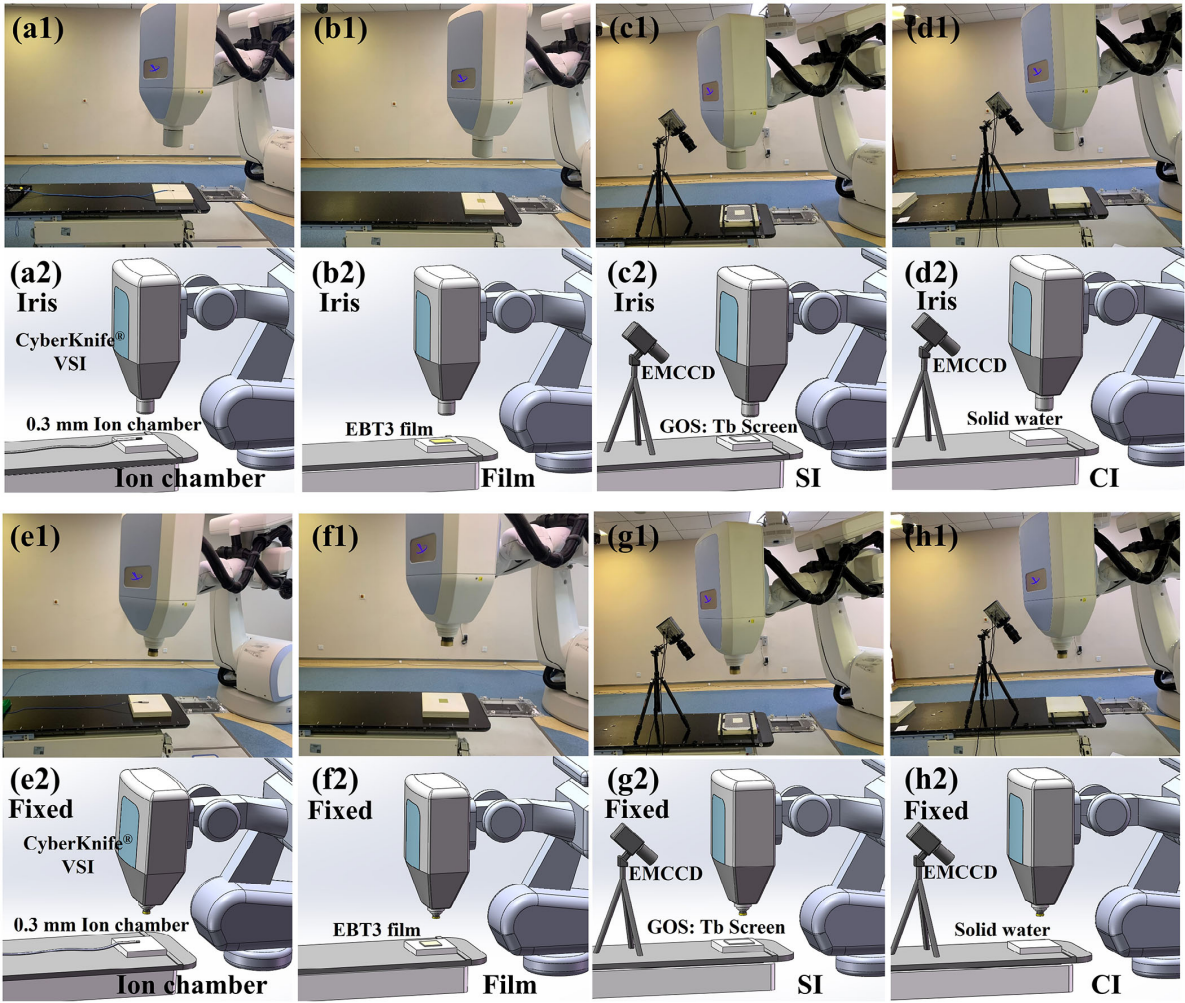
Actual scene and schematic of the experimental setup. Actual scene of ion chamber, film, SI, and CI measurements for iris (a1–d1) and fixed circle (e1–h1) fields. Corresponding schematic of ion chamber, film, SI, and CI measurements for iris (a2–d2) and fixed circle (e2–h2) fields.

All radiation fields were delivered using the CyberKnife® VSI Robotic radiosurgery system (CNNC Accuray) operated at a dose rate of 1000 MU/min for photons. Beams of 6 MV photons were used to irradiate the solid water, GOS: Tb scintillation screen (YuGuang New Material Technology Co., Ltd, Shanghai), ionization chamber (PTW, Semiflex, Freiburg), and EBT film (Gafchromic, Bridgewater, NJ) at an SSD of 800 mm, respectively. The actual scene and schematic of the experiments are illustrated in Figure 1. The experiments included the following four parts.

Part 1: Dose‐response and sensitivity of CI and SI to changes in CyberKnife® output was compared to an ionization chamber. To assess dose response, the relationship between the optical intensity and the delivered dose was evaluated on solid water for delivered doses of 200, 400, 600, and 800 MU. Started from 100 MU, the MU was continuously changed by ± 1%, ± 3%, ± 5%, and ± 7% for all measurement systems with a field of 60 mm for fixed cones and iris collimators. These processes were repeated three times, and the standard deviation was quantified as measurement uncertainty.

Part 2: Measurements for beam shape. For each measurement imaging scintillation and Cherenkov emission, a total of 200 MU were delivered per field. And 500 MU were used to maximize the signal contrast on each film. Beam shapes were using the fixed cones and iris collimators, setting the beam sizes of 10, 15, 20, 30, 35, 40, 50, and 60 mm.

Part 3: To evaluate the sensitivity of the CI and SI to small beam position variation, 40 mm circle and iris fields were delivered at a known starting position. The images obtained from this position were designated origin images and used for all shift comparisons. Field shifts with known introduced distance were performed using the treatment couch motion, and shift distances of 1, 3, and 5 mm were tested. Beam shapes were measured using SI, CI, and the film.

Part 4: To evaluate the beam measurements reproducibility with CI and SI, the reproducibility of the entire procedure, in particular imaging settings and reference image acquisition was investigated by measuring the beam shape of the 40 and 60 mm circle and iris field for three times on 5 consecutive days.

### 2.2 Image acquisition and processing

Each beam was imaged using a tripod‐mounted EMCCD camera (Andor iXon ultra‐888), and a 40 mm f/1.8 Canon lens was equipped for adjusting focus within a specific range. The camera was placed at the base of the couch at a 75 cm distance between the image sensor and the imaging center. To avoid the influence of ambient background light, all indoor lighting was blocked during CI and SI measurements. Considering the SNR and after a series of adjustments, the exposure time was determined to be set as 0.01 and 0.15 s in SI and CI, respectively. Background image with the same exposure time was acquired before each beam output, and it was subtracted from each frame of CI and SI. Dark‐field and flat‐field response corrections were used for all images to reduce the light response differences between image sensors. Two filtering methods were adopted to eliminate noise caused by stray radiation: (1) 3‐image temporal median filtering, in which the median value for each pixel was calculated for three consecutive images acquired at times t1, t2, and t3 to produce a new image comprising these median values. This guaranteed that pixel values falling far outside the norm would be eliminated from the data set. (2) 3×3‐pixel spatial median filtering, in which the value of each pixel of the original image was replaced by the median value computed over a 3×3 square window.

**FIGURE 2:**
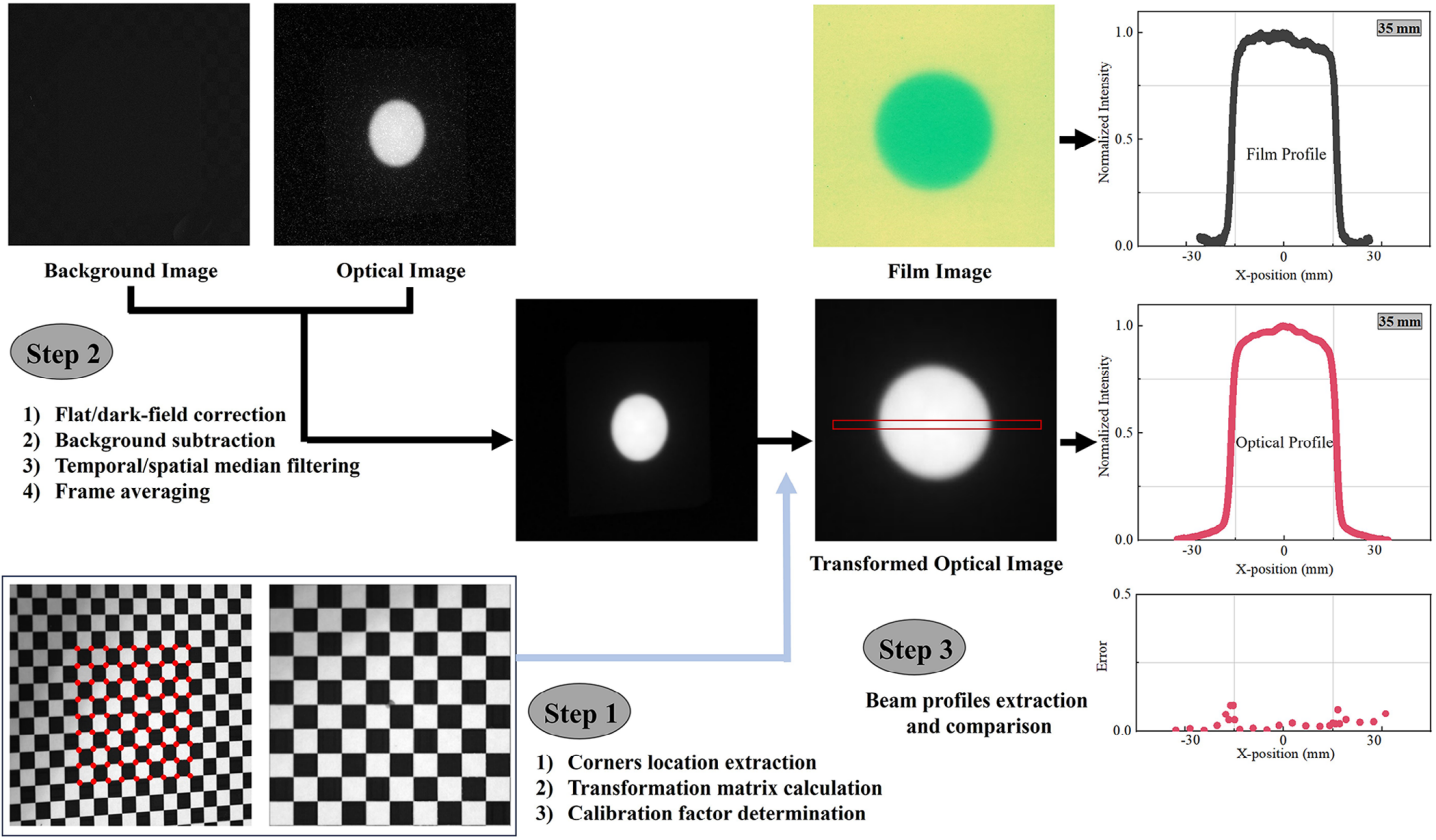
The flowchart of the image‐processing described in Section 2.2. Step 1: The square patterned board used for perspective transformation was illustrated, the corners detected by the OpenCV library were marked with red dots, and the image was remapped to project an undistorted view of the board. The pixel calibration factor was also determined in this step using the known size of checkerboard patterns. Step 2: The raw frames were post‐processed with four procedures: flat/dark‐field correction, background subtraction, temporal/spatial median filtering, and frame averaging. Step 3: The beam profiles were extracted from transformed optical images and EBT3 film and then compared.

To perform perspective transformations of acquired images to simulate the beam's eye view, the matrix used for image transformation was verified using checkerboard patterns.^27^ The checkerboard patterns chosen for our research consist of 50 black and 50 white cells. The OpenCV checkerboard function was applied to extract pixel locations of the corners in the image, as shown in Figure 2.^35^ Based on the extracted points above, the perspective transformation was used to translate distortion images to the expected main view. And then, applied this correction to all SI and CI images obtained from our experiments to achieve the beam shape measurements comparison with film. To minimize the profile error caused by optical scattering, a maximum slope profile estimate was used to determine the boundaries of scintillation and Cherenkov emission. Maximum slope profile estimate was defined by the extrema points of the first derivative of the profile line. When selecting the maximum slope point, we retained three decimal places of the corresponding abscissa of the fitted profile curve. To obtain a proper pixel size, an image of the checkerboard at 800 SSD was acquired. Perspective transformation as outlined above was applied to this image. Using the known size of checkerboard patterns, we determined the pixel calibration to be 0.64 mm/pixel.^36^ An overview of image processing procedures is illustrated in Figure 2.

## 3 RESULTS

### 3.1 Dose‐response and output sensitivity

**FIGURE 3:**
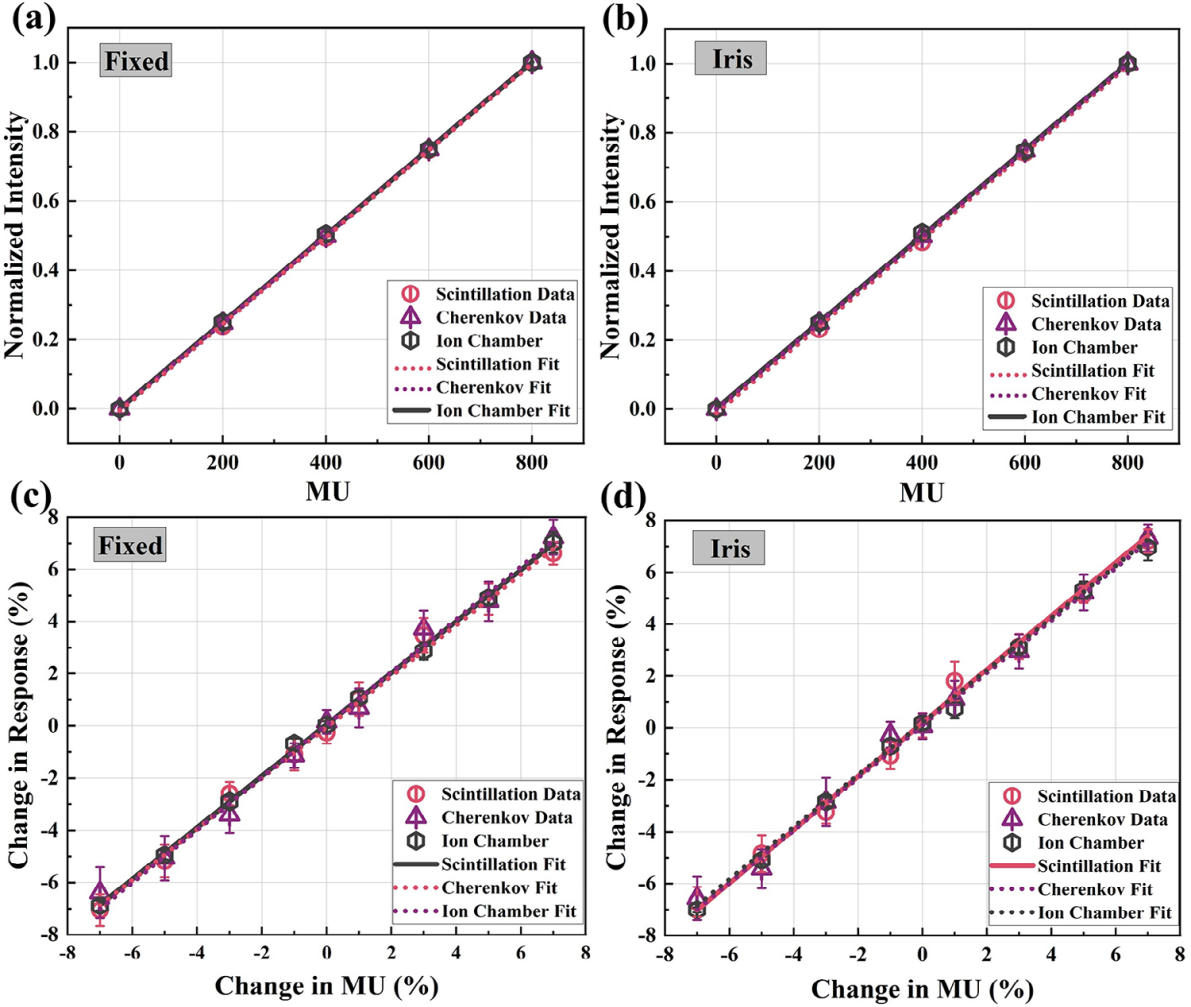
(a) Normalized intensities measured by SI, CI, and ionization chamber as a function of the MU at circle (a) and iris (b) field, respectively. Sensitivity of Cherenkov and scintillation intensity compared with ionization chamber charge collection to small changes in CyberKnife® output at circle (c) and iris (d) field.

An average pixel count from a 20 × 20 pixel ROI in the image surrounding the center of the field is used as a measure of Cherenkov intensity, scintillation intensity and this reading along with the ion chamber reading are both normalized to their respective values at 100 MU. The average optical intensity collected at the circle field and iris field is found to be linear with MU, as shown in Figure 3(a),(b). Figure 3(c),(d) shows a comparison of response sensitivity to small deviations in CyberKnife® beam output for an ionization chamber and these two optical imaging methods. Even with a 1% (1 MU) undulate in dose output, the optical counts from CI and SI exhibit high response sensitivity and linearity within the dose variation range of 1−7 MU, no matter for iris and circle filed (*R* ^2^ is all above 0.995). The standard deviation of three continuous measurements is displayed on error bars.

### 3.2 Beam shape measurements

**FIGURE 4:**
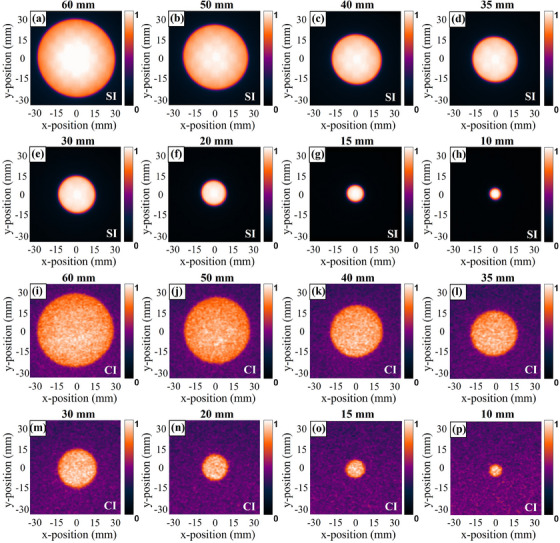
Transformed scintillation (a–h) and Cherenkov (i–p) images for various circle field sizes. Both optical and film images are normalized to their maximum pixel value, respectively.

**FIGURE 5:**
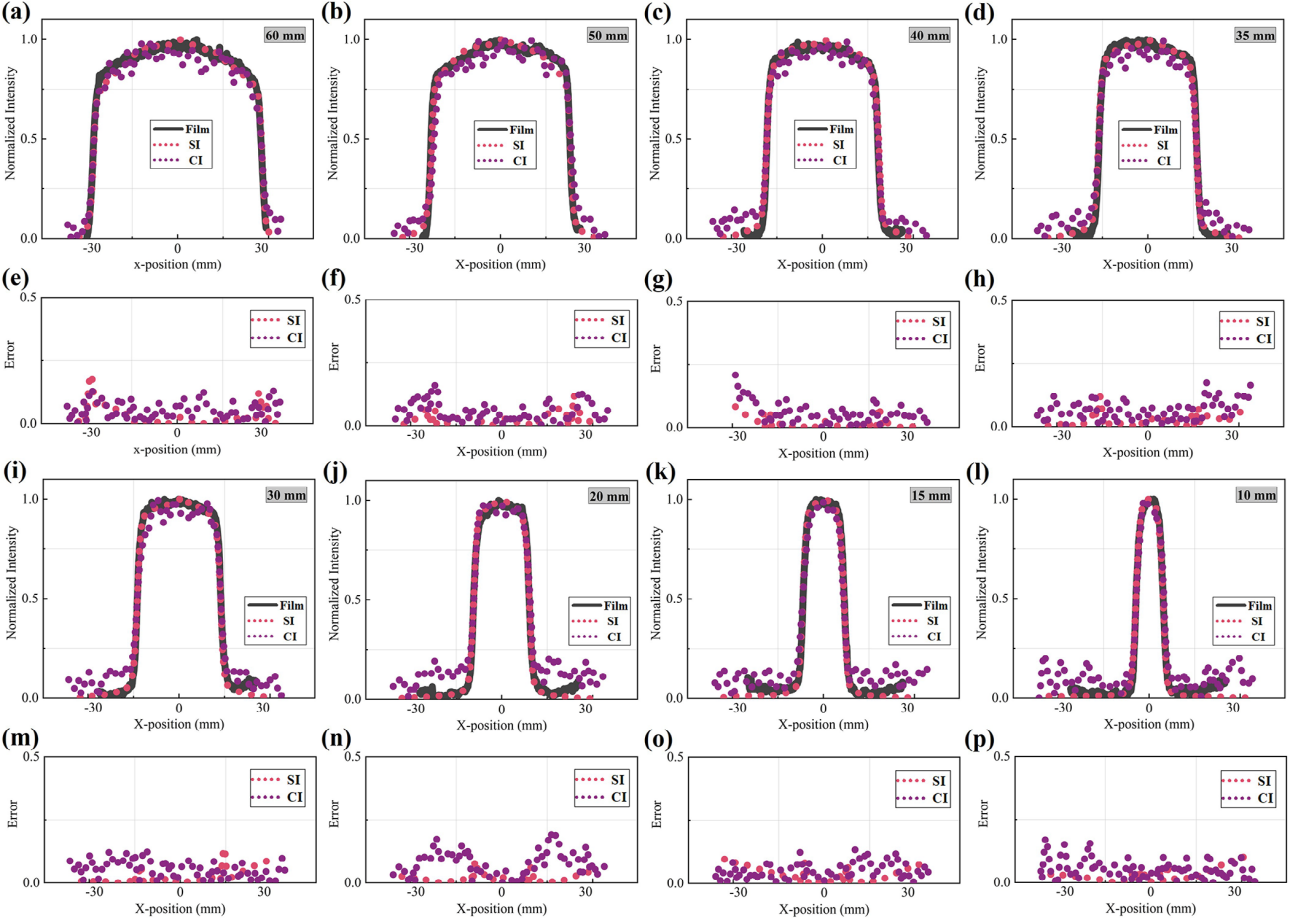
(a–d, i–l) X‐direction profiles measured from SI, CI and film measurement for circle field. The solid gray line represents the film profile data, the purple and red dots represent the Cherenkov and scintillation data, respectively. (e–h, m–p) represent the error between the film fitting curve and the optical data.

**FIGURE 6:**
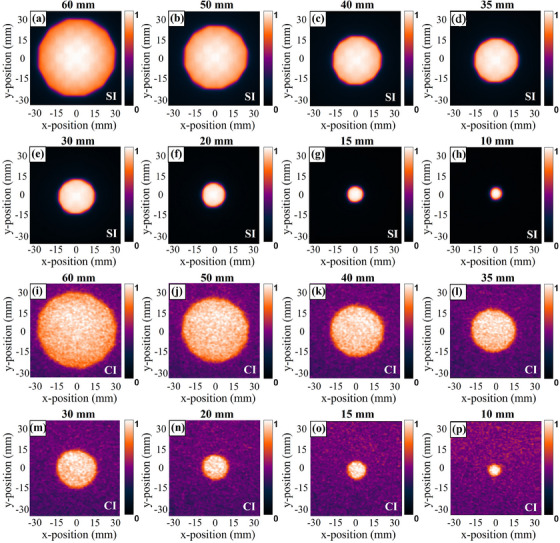
Transformed scintillation (a–h) and Cherenkov (i–p) images for various iris field sizes. Both optical and film images are normalized to their maximum pixel value, respectively.

**FIGURE 7:**
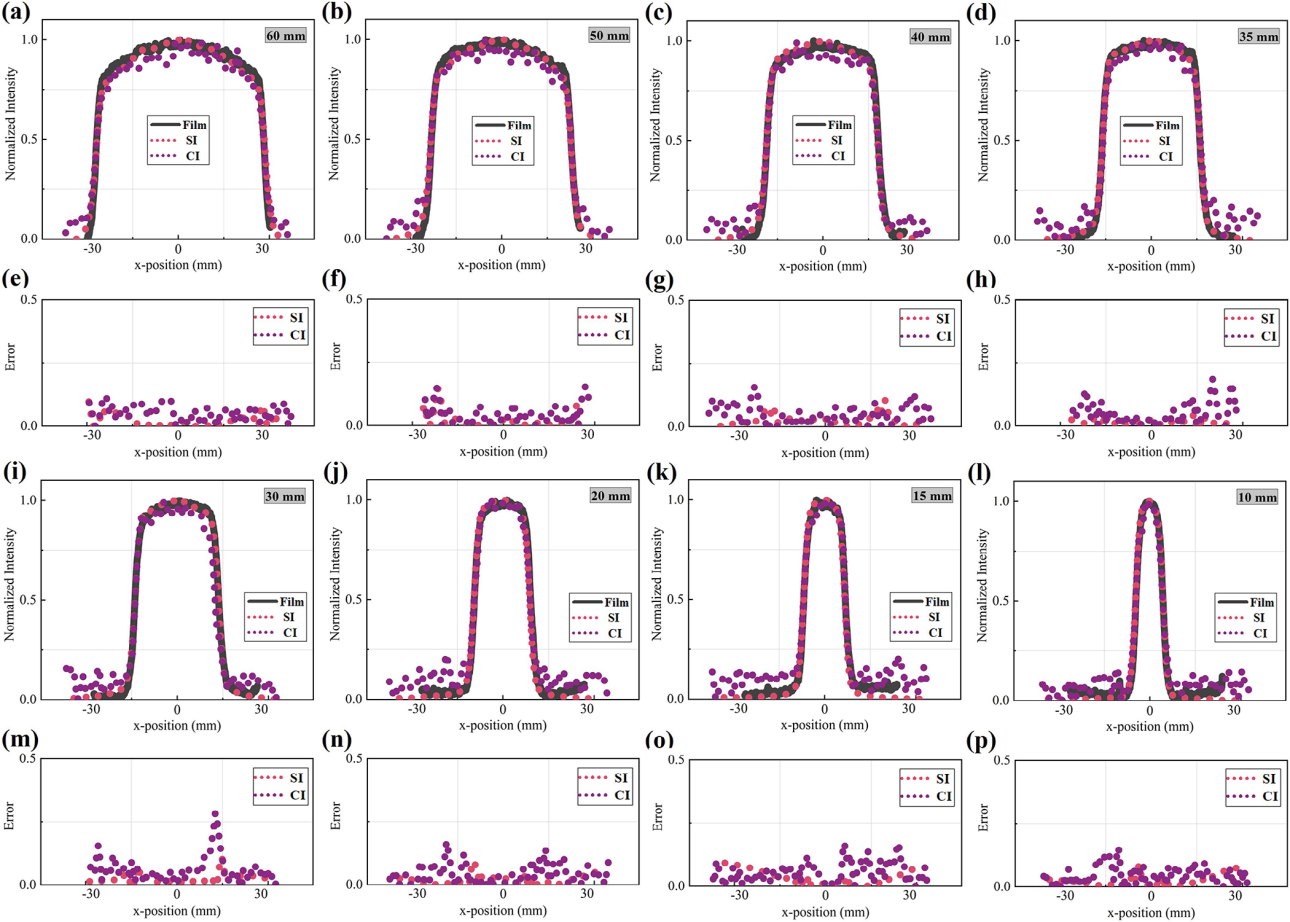
(a–d, i–l) X‐direction profiles measured from CI, SI, and film measurement for iris field. (e–h, m–p) represent the error between the film fitting curve and the optical data.

Figure 4 shows the transformed beam shapes measured by SI and CI for the eight different circle field sizes tested. The field profile is extracted from 100 × 5 pixel ROI selected from the middle of the radiation field as shown in Figure 2. It is obvious that the circle and iris beam‐shape measurements of SI and CI all exhibited considerable agreement with the film. Both x‐ and y‐direction profiles are measured for the circle field, and x‐direction profiles of SI and CI are shown in Figure 5(a–d) and (i–l) along with film profiler measurements for each circle field size. Figure 6(e–h) and (m–p), illustrate the difference between the measured profiles of the optical imaging method and film. The profiles along opposite directions on both sides are measured for the iris field, corresponding results are shown in Figures 6 and 7.

The results of measurements are summarized numerically in Table 1, listing the width from SI, CI, and film measurements for circle and iris fields. The profile boundaries are determined by the maximum slope profile estimate to reduce the impact of noise.^26^ For the circle fields, the average measurement discrepancy between the SI and film profiles is 0.09 mm (0.38%) in the x‐direction and 0.24 mm (0.96%) in the y‐direction. The corresponding discrepancy measured by CI and film is 0.17 mm (0.86%) in the x‐direction and 0.29 mm (1.1%) in the y‐direction. For the iris fields, using film measurements as a reference, the beam profile measured by SI has a margin of error of 0.2 mm (1.13%), and for CI, it is 0.29 mm (1.33%).

**TABLE 1:** Results of beam size measurements as measured from SI, CI, and film.

				Discrepancy			
	Measured size			SI		CI	
Beam size	SI	CI	Film	mm	%	mm	%
X‐direction							
10	9.78 ± 0.08	10.12 ± 0.21	9.85	−0.07	−0.71	0.27	2.74
15	14.96 ± 0.19	14.95 ± 0.09	14.86	0.10	0.67	0.09	0.61
20	20.12 ± 0.05	20.44 ± 0.36	20.04	0.08	0.4	0.40	2.00
30	30.24 ± 0.25	29.97 ± 0.17	30.12	0.12	0.4	−0.15	−0.5
35	34.90 ± 0.46	34.88 ± 0.35	35.08	−0.18	−0.51	−0.2	−0.57
40	40.36 ± 0.12	40.46 ± 0.41	40.42	−0.06	−0.15	0.04	0.10
50	50.65 ± 0.2	50.58 ± 0.54	50.71	−0.06	−0.12	−0.13	−0.26
60	61.11 ± 0.42	61.18 ± 0.71	61.14	−0.03	−0.05	0.04	0.07
Y‐direction							
10	9.62 ± 0.28	9.92 ± 0.39	9.74	−0.12	−1.23	0.18	1.85
15	15.21 ± 0.47	15.07 ± 0.35	14.84	0.37	2.49	0.23	1.55
20	20.48 ± 0.17	20.57 ± 0.63	20.19	0.29	1.44	0.38	1.88
30	30.48 ± 0.59	30.54 ± 0.55	30.79	−0.31	−1.01	−0.25	−0.81
35	35.83 ± 0.18	35.45 ± 0.47	35.79	0.04	0.11	−0.34	−0.95
40	40.58 ± 0.4	40.78 ± 0.58	40.67	−0.09	−0.22	0.11	0.27
50	51.18 ± 0.4	50.62 ± 0.82	50.94	0.24	0.47	−0.32	−0.63
60	60.82 ± 0.37	60.74 ± 0.63	61.24	−0.42	−0.69	−0.50	−0.82
Opposing‐sides							
10	10.32 ± 0.23	10.18 ± 0.31	9.77	0.55	5.63	0.41	4.20
15	15.16 ± 0.47	14.89 ± 0.55	15.28	−0.12	−0.79	−0.39	−2.55
20	19.84 ± 0.34	19.81 ± 0.18	20.05	−0.21	−1.05	−0.24	−1.20
30	30.74 ± 0.28	30.57 ± 0.73	30.69	0.05	0.16	−0.12	−0.39
35	35.93 ± 0.52	35.74 ± 0.29	35.80	0.13	0.36	−0.06	−0.17
40	40.88 ± 0.19	40.96 ± 0.36	40.74	0.14	0.34	0.22	0.54
50	50.97 ± 0.41	51.03 ± 0.44	50.73	0.24	0.47	0.30	0.59
60	61.10 ± 0.36	61.54 ± 0.57	60.94	0.16	0.26	0.60	0.98

### 3.3 Sensitivity test of field position variation

**FIGURE 8:**
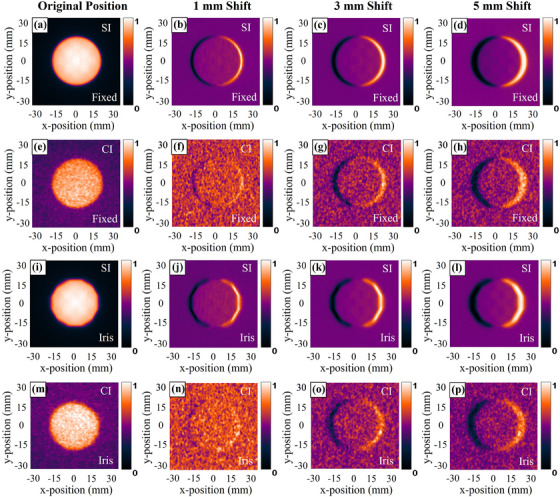
The original images for 40 mm field (left‐most column) and the difference maps associated with lateral shifts over distances of 1, 3, and 5 mm, respectively.

As shown in Figure 8, analysis of all shifted images for the 40 mm circle and iris field is performed by creating difference maps for each shift. Obviously, the sensitivity of the SI and CI allows the detection of small field variations even for a 1 mm shift. Table 2 provides a summary of the known and measured shift using SI and CI, the measured shift distance is calculated based on the profile difference between the shifted and original image. The mean differences in shift distance measured by SI are equal to 0.11 mm for the circle field and 0.2 mm for the iris field. Corresponding mean differences measured with the CI are 0.26 mm and 0.35 mm, respectively.

**TABLE 2:** The known and measured shift using SI and CI.

	SI shift (mm)		CI shift (mm)		SI error (mm)		CI error (mm)	
Known shift distance	Circle	Iris	Circle	Iris	Circle	Iris	Circle	Iris
1 mm	1.14 ± 0.48	0.79 ± 0.77	1.22 ± 0.73	1.45 ± 0.52	0.14	0.21	0.22	0.45
3 mm	3.14 ± 0.24	2.76 ± 0.19	3.4 ± 0.62	3.39 ± 0.81	0.14	0.24	0.40	0.39
5 mm	5.05 ± 0.33	4.85 ± 0.48	5.15 ± 0.89	4.78 ± 0.54	0.05	0.15	0.15	0.22

### 3.4 Reproducibility test of beam measurement

Beam shape size was measured for the circle and iris field with 40 and 60 mm cones collimator for three times on 5 consecutive days. The results of measurements are shown in Table 3, listing the mean width from SI, CI, and film measurements for circle and iris fields over 5 days. The measured standard deviation (SD) of SI for the 40 mm circle field is 0.76 mm (1.9%), corresponding SD measured by CI is 0.89 mm (2.2%). For the iris fields, the beam size measured by SI has a SD of 0.32 mm (0.8%), and for CI, it is 0.86 mm (2.2%).

**TABLE 3:** Results of beam measurement and its standard deviation.

	Day 1	Day 2	Day 3	Day 4	Day 5	SD
Circle						
40‐SI	40.73 ± 0.13	40.86 ± 0.37	40.54 ± 0.48	40.72 ± 0.57	39.04 ± 0.71	0.76
40‐CI	40.91 ± 0.27	40.77 ± 0.31	39.07 ± 0.52	40.71 ± 0.22	39.27 ± 0.5	0.89
60‐SI	60.11 ± 0.46	60.34 ± 0.88	60.88 ± 0.32	60.47 ± 0.54	60.34 ± 0.27	0.28
60‐CI	60.28 ± 0.83	60.53 ± 0.2	60.99 ± 0.17	61.03 ± 0.69	60.15 ± 0.54	0.4
Iris						
40‐SI	41.13 ± 0.34	40.25 ± 0.64	40.74 ± 0.88	40.6 ± 0.43	40.78 ± 0.35	0.32
40‐CI	40.34 ± 0.58	41.31 ± 0.55	39.22 ± 0.31	40.18 ± 0.27	41.26 ± 0.54	0.86
60‐SI	60.33 ± 0.4	60.85 ± 0.33	60.94 ± 0.19	59.38 ± 0.79	59.56 ± 0.33	0.72
60‐CI	60.91 ± 0.74	61.41 ± 0.56	59.45 ± 0.45	59.27 ± 0.67	60.74 ± 0.61	0.94

## 4 DISCUSSION

This study assessed and compared the effectiveness of scintillation and Cherenkov imaging in quantitatively measuring CyberKnife® beam characterization as part of routine quality assurance (QA). Output sensitivity, beam shape, position variation sensitivity, and reproducibility measured by SI and CI systems are comparatively discussed with traditional measurement methods. The first objective of this work is to measure the dose‐response output sensitivity using SI and CI. The proportionality between dose and optical intensity is a commonly pursued dosimetry characteristic as it facilitates easy dose deposition analysis. The overlap of these three fitting lines reflects the consistencies of the response sensitivity between the two optical imaging methods and the ionization chamber (Figure 3c,d). Both SI and CI show a sensitive response to MU change. Our pre‐experiment indicates that the difference in optical intensity between capturing Cherenkov emission independently and recording scintillation and Cherenkov simultaneously is up to three orders of magnitude. Therefore, Cherenkov emission from scintillation screen is negligible for the research of scintillation imaging. Certainly, determining the emission spectra of Cherenkov emission and scintillation emission at the CyberKnife® irradiation surface is significant to sort out the composition of optical emission and further overrule the potential effect of both emission on each other result.

The second objective of this work is to quantitatively evaluate the accuracy of the surface beam shape measurement using SI and CI. As can be seen from Figures 5 and 7, the circle and iris beam profile measurements of SI and CI all exhibited a good consistency with the film. It is obvious that the disagreements are largely concentrated in the penumbra region, and the discrepancy between CI and film is significantly higher than that between SI and film. This is mainly ascribed to two reasons. First, the dose fell sharply in the penumbra region, but the optical blurring affects the falloff steepness of the optical profile. Second, to detect the weaker Cherenkov signal, the exposure time for CI is set much higher than SI. Therefore, more noise caused by stray radiation also affects profile measurements. Based on the above analysis, we believe that the SI can be considered a priority optical method for beam shape measurement in routine QA of CyberKnife®. To minimize the profile error caused by optical scattering, we also tested FWHM and maximum slope profile estimate to determine the boundaries of scintillation and Cherenkov emission. The FWHM is estimated using the maximum and minimum signal values along the profile line, which are vulnerable to stray radiation. Combining our tests with the study of Miao et, al. the second definition for profile edges is used as an evaluation criterion, and all fields with results in Table 1 are calculated using the maximum slope profile estimate.^26^ When compared to the film measurements, the SI measurements are generally within CyberKnife® QA guidance tolerance (with the greater values of 0.18 mm or 0.71% in x‐direction, 0.42 mm or 2.49% in the y‐direction for circle fields, and 0.55 mm or 5.63% for iris fields). Similarly, CI measurements are also within this tolerance, corresponding maximum error is 0.27 mm or 2.74% in the x‐direction, 0.38 mm or 1.88% in the y‐direction for circle fields, and 0.60 m or 2.55% for iris fields. It is noteworthy that the honeycomb patterns in some scintillation images as shown in Figures 4 and 6. We attribute the honeycomb patterns of scintillation images to the potential optical emission from transparent acrylic checkboard. In the part of scintillation imaging, the GOS: Tb scintillation screen is placed directly on the checkboard to maintain a fixed relative position between the camera and the imaging plane. The scintillation of acrylic resin might have a slight impact on the distribution of optical emission within the beam field. However, the purpose of our research is to provide a high‐resolution method for CyberKinfe® beam shape measurement. Due to its independence from absolute dose measurement, the partly inside inhomogeneous will not affect field profile (beam shape) measurements with the use of a maximum slope profile estimate. This problem can be improved through adding a black opaque backing to the scintillation screen during the next work to obtain absolute dosimetry with a fixed geometry calibration.

The third objective of this work is to assess the sensitivity of SI and CI for field position variation and beam measurement reproducibility. The pixel density of SI and CI provides high resolution in measuring small shifts in field position. Table 2 demonstrates the consistency between SI, CI shift, and known shift for small field position shifts, being less than 0.24  and 0.45 mm, with the ability to detect a 1 mm shift for the circle and iris field. Obviously, SI can sensitively discriminate small field shifts comparable to known shifts, without a time‐consuming post‐processing process like film. Although more noise and scattering caused CI to not work quite as well, it is still accurate and can be improved by synchronizing the camera shutter with radiation pulses by ICCD. The beam size deviation that occurred in reproducibility test (as shown in Table 3) is mainly ascribed to three reasons. First, it needs to be taken into account that the scattered megavoltage x‐ray photons are most likely to penetrate the camera exterior to CCD sensor resulting in hotspots in the recorded image, which may affect profile extraction due to their non‐localized and random nature. Second, even though we have marked the positions of the camera and solid water cautiously, daily manual adjustments still introduce errors in their relative positions. Third, the unstable ambient light detected each day (although shading has already been applied) also impacts optical images after background deduction. In future studies, we plan to securely package the CCD camera and imaging board in a specially designed dark box to ensure that the relative position of the CCD camera and imaging board remains invariant and further reduce the impact of uncertain light sources in the environment. Meanwhile, the shield will also be considered to be added on both sides of the CCD camera to minimize the impact of stray radiation.

Compared with scintillation screen is needed for SI, additional imaging media is not required for CI, which allows CI to have unique advantages during field imaging. First, Considering the operation life and radiation damage of scintillation screen, the stability of the CI system is inevitably higher than the SI system in the long term. Of course, the actual long‐term stability of SI and CI will be experimentally validated in our following study. Second, imaging Cherenkov photons emitted from the surface of patient during CyberKnife® radiotherapy would enable real‐time verification of in‐patient targeting and treatment delivery accuracy.^37^ If Cherenkov imaging is further applied to real‐time CyberKnife® dosimetry, exploring the intra‐ and inter‐patient variability in superficial tissue types is essential. Hachadorian et al. investigated the potential to correct superficial vasculature using spatial frequency domain imaging to map tissue optical properties across large fields of view.^38^ And then, they corrected Cherenkov intensity by integrating x‐ray CT to mitigate dependencies on intrinsic tissue optical properties.^39^ In the next work, SI and CI techniques will be performed for MLC positioning accuracy verification like picket fence test and beam parameter checks comprising symmetry, flatness constancy, and penumbra for CyberKnife® M6 system. Exploring the application of CI in real‐time beam visualization during CyberKnife® radiotherapy to detect small deviations in patient positioning and intra‐fraction anatomical movements is undoubtedly significant and this is also within our considerations.

## 5 CONCLUSION

The practicability of SI and CI for CyberKnife® radiation beam shape verification in real‐time is assessed in this study. Output sensitivity, field profiles and position variation sensitivity, and beam measurement reproducibility measured by SI and CI systems are comparatively analyzed with an iron chamber and EBT3 film. This work indicates that CI and SI could also be used to measure field profiles and detect small field position variations with comparable accuracy to film, however had the advantage of instantaneous readout and simplified data collection. The first application of the CI technique for beam shape measurements in routine QA of CyberKnife® is tested. Meanwhile, the systematic comparison of SI and CI also provides a suitable reference for the measurement methods selection in CyberKnife® QA.

## AUTHOR CONTRIBUTIONS

**Fengwei Cui**: Data presentation and visualization; writing—reviewing and editing. **Tao Jin**: Experiment conduction; formal analysis; writing—original draft. **Mingzhu Li**: Data curation; writing—original draft. **Lei Zhu**: Writing—original draft. **Xing Di**: Conceptualization; methodology; writing—reviewing and editing; supervision. **Huaguang Zhu**: Conceptualization; writing—reviewing and editing; supervision.

## CONFLICT OF INTEREST STATEMENT

The authors have no relevant conflicts of interest to disclose.

## Data Availability

The data supporting the findings of this study are available from the authors upon reasonable request.
